# Our Cantata

**Published:** 1881-07

**Authors:** Ausburn Towner


### OUR CANTATA.

**Final Passages from the Records of a Musical Society.**

AUSBURN TOWNER.

We were the Erato Club of Niskayuna, not the Erratic, as some called us, and I was the president, director and manager. We had incurred some indebtedness during a brief existence, and determined to make a bold plunge to cancel it. We would give the Cantata of "Belshazzar," the music of which was attractive and the general character of the piece quite on the spectacular order. We rehearsed carefully for a number of weeks, and the jealousies and heart-burnings consequent upon the distribution of the solo parts gradually faded away, giving place in the minds of the hundred or more persons chosen for the chorus, to a hearty and genuine interest in the success of the entertainment. Unfortunately we had not had a dress nor a stage rehearsal at all, because on the night appointed for that purpose a traveling show unexpectedly occupied the hall where we were to sing. But the management of the stage seemed to me the very easiest part of the whole undertaking, and I gave myself no apprehensions in regard to it.

Neither could we prevail upon the German orchestra, who were to do the instrumental part of the evening, to meet when we met and rehearse the whole of the piece. The stout leader shrugged his shoulders when he looked at the score, and said with a sort of sneer: "Mine child dere can play him on sight." So I had no apprehensions about that part, and, besides, a very competent young lady pianist, who had accompanied us in all our rehearsals, could be safely relied on.

A very large audience assembled on the night of the entertainment. When I put in my appearance the hall was crowded, up stairs and down, with most of the best and first citizens of Niskayuna and their families, representing enough money to cancel all of our indebtedness and leave a handsome balance in our treasury besides. Every one behind the curtain was in the best of spirits. All of the ladies were delighted with their really beautiful dresses, and the gentlemen were by no means dissatisfied with theirs. The very first disturbing event that clouded my anticipations, and the first one also that had occurred since the conception of the undertaking, came to me as I stepped out from my dressing room, clothed in the gorgeous apparel of Belshazzar's Master of Ceremonies. Our Cyrus was indisposed and missing! He had been having a little preliminary skirmish with John Barleycorn, and John had done for him what it had been expected he would have done for Belshazzar—floored him. After a hurried and nervous consultation, it was decided to promote the leader of one of the files of Cyrus's army of twenty-four men, to fill the place of Cyrus himself, to exchange the clothes of a common soldier for the glittering armor and white plumed helmet of the general! He had heard the part rehearsed so frequently that he knew it quite as well as the one who had been originally cast for it. This would, of course, somewhat endanger the efficiency of the troops, and materially interfere with the accuracy of some brilliant evolutions and manœuvres in which they had been drilled for a number of weeks. But the chance had to be taken.

This enforced alteration occupied considerable time, and it was twenty minutes after eight when the curtain at length rolled up, the audience meanwhile having grown very impatient, making the hall resound with their calls for a beginning, and some quite inappropriate and unnecessary ones from the gallery, which had allusion to what they inaccurately termed a "rag" in front and to the capabilities of the chief character being able to make a satisfactory appearance on a race track.

We had used the whole stage for the first scene, and it went off splendidly, the chorus being in fine voice, and winning the hearty and sincere applause of the audience. The delay seemed to have been forgotten and forgiven. The second scene was to have been an interior and needed some considerable preparation. Just before the end of the first this idea struck me, and I didn't know what to do. We could not prepare the stage in the face of the whole audience. I ran to the head scene shifter and appealed to his larger experience. While I was seeking him the chorus came to an end, the people all retired and the stage was waiting. I found him in a corner, intimately concerned with a small black flask. His only reply was that the "plot" of the stage had been furnished him and he knew his business "better'n any amateur." I don't like to say what kind of an amateur he mentioned, for although the word he used sounded like something that had reference to water privileges, it didn't mean that, but something quite the opposite. Nevertheless I hurried him to his work as well as I could, and presently I heard the rolling of the flats as they were pushed out in the first groove, hiding from the audience nearly the whole stage, while it was being prepared for the second scene The sliding of the rollers was followed by a strange noise in front. It sounded like a sudden and fierce summer shower. First, a little titter ran around the house, like the patter of a few advancing drops, then a great roar of irrepressible laughter and the clapping of a multitude of hands. I rushed to the prompt desk to see what was the matter, and for a moment stood dumb with a rather amused astonishment. One flat of the scene was the half of a very pretty English cottage, the other flat was a dense forest. They matched about as well as a donkey's head on a man's shoulders, and were so utterly inharmonious that the effect was overwhelmingly ridiculous. I could not help laughing, in spite of an agony that covered my body with a cold perspiration. To make the matter worse, some one suddenly pushed a half of an archway interior part way over the forest scene, complicating the view and giving it an appearance of the upper part of a room in the tops of the trees; and at the wings, on one side was a handsome panel parlor, while on the other stood out in bold relief a great tree. It was useless to try and rectify these errors, so I hurried up the preparations behind all that I could, but it seemed as if we would never get ready and that the audience would never stop laughing. Besides, how could they ever appreciate the beautiful music of the Cantata, which I was sure we could render in capital style if they got in such a gale of merriment, ready to explode at the slightest mistake or balk? Then there suddenly sprang into my mind the favorite Limerick saying or proverb, "Single misfortunes never come alone," an expression that was abundantly verified before the evening was over.

But finally the stage was ready and the flats were pulled back. The scene was mostly made up of solo performances by people representing certain ones of Belshazzar's court, and they all acquitted themselves with so much credit, especially the Queen, who was a woman of beautiful person and the soprano of the most popular church in town, that my fears and apprehensions for the moment all vanished, and I felt assured that the remainder would go along with perfect smoothness.

This very comfortable state of mind didn't last long. The end of the scene and the act was approaching, and the prompter had rung to warn the men at the curtain to be ready to let it down. The five singers on were so far forward that if the curtain came down it would fall behind them. I tried to attract their attention to the fact by snapping my fingers vigorously, as I stood at the first entrance, but none of them paid any attention to the signal except the stout baritone, who had "been there before." He looked upwards, and seeing the situation stepped back far enough to clear the curtain when it came down. None of the others followed him or seemed to know what he was about, and my agony became intense. I shrieked, in a stage whisper, that I am sure was loud enough to be heard all over the house, for an audible titter followed its utterance, to "get back," but that, also, was unheeded. The stout baritone stepped forward again, and catching hold of the alto, pulled her back by main force, all the while looking upward, plainly announcing to the amused audience, in this manner, what the matter was. At that moment came the signal for the curtain, and down it commenced to roll. My agony became terror. If the heavy roller should continue to descend it would surely strike the soprano, who was singing with all her might, square on the head, and would shut the tenor out. It could not have been more than a foot above them, she, meanwhile, all unconscious of her peril, when I sprang to the bell, gave the signal for rising, and the motion suddenly reversed, up went the cloth again. The singers just then fell back behind the line, and with a signal for a quick drop, the curtain descended on the first act, amidst the roar of the audience, the laughter entirely drowning the applause.

We could get no music from the orchestra between the acts, because, as I learned afterward, when we were making arrangements with them nothing had been said about their playing then, and they always "stood by the letter of their contract." Consequently, the waits were tedious, hurry as I might the stage preparations, these being by no means the least annoying omissions or commissions of the evening.

After a while we were ready for the second act, in which appeared the Jewish chorus. The signal was given for the music and the curtain. I heard a few turns of the wheels in the flys; then they stopped and there was another roar from the audience. I rushed again to the prompt desk to see what was the matter. The curtain was up only about three feet from the floor, and the whole Jewish chorus were standing about the stage, convulsed with laughter. Hurriedly I sought the cause. Right in the center of the stage, and just by the curtain, there stood one of the chorus singers, backed up against the roller and making the most frantic exertions. I can hardly say stood, either, for he was drawn up so that the tip ends of his toes touched the floor, and he was throwing his arms about like an inexperienced swimmer. He had been standing with his back to the curtain, and his long, loose robes had been caught by the roller in its sudden ascent, and it had gone up as far as it could go, revealing to the audience, in the glare of the footlights, a solitary pair of exceedingly slim legs clothed in tights considerably too large for them, and nothing else, the legs meanwhile flying about like the easy jointed limbs of a dancing jack. I heard one shrill voice call out from the front, "Shoot them legs," another "Give those calves more bran," and another, "Hallo, Mr. No-body." It was exasperating, simply exasperating; and I certainly didn't hurry to have the foolish and careless fellow extricated from his uncomfortable position. I let the audience enjoy the fun for some minutes before I cleared the way, and sent up the curtain. They didn't get over the display for the whole act, and probably would not have recovered from it during the whole evening if other things still more ludicrous had not occurred afterwards. As, at the end of that same scene, the flats were to close on a tableau with red fire. They did close, and the wing on one side was not pushed out far enough to hide the man standing there in his shirt sleeves holding his pan of burning chemicals. He got a round of applause that he little expected, and quite as tumultuously given as that showered upon myself in the second scene of this act. In setting the stage, two chairs had by some means been forgotten, upon which the alto and the little girl who played the part of her daughter had, by the exigencies of the scene, to be seated. I saw the omission, and just before the chairs were to be used I rushed upon the stage with them, placing them where they should be. I, the master of ceremonies of Belshazzar's court, acting as waiter for a Jewish woman and her child! The audience caught the inaptitude and unfitness of the proceeding and gave it to me for it with a round of cheers.

The third act opened in the camp of Cyrus, with Cyrus himself down in front, asleep upon a bank. Two back flats were pushed apart in representation of a dream visiting the general, disclosing an angel upon a pedestal, who sings one of the sweetest melodies in the Cantata. The angel was represented by a beautiful little maiden, daughter of one of the first men in Niskayuna. She was dressed in a gauzy material that made her look very spiritual and dream-like, and sang her song charmingly. She was heartily applauded when she concluded. I began to think that my troubles were ended. I saw Cyrus rise from the bank and the business of the piece go on smoothly for a moment or two, and then came again that ominous titter from the front. "Good Heavens!" I exclaimed, "what can possibly be wrong now?" I looked from one of the wings up the stage, and to my consternation, there still stood the angel, with her white arm outstretched, in full view of the audience, the dream-illusion entirely dissipated. The flats that were to have shut her from sight at the conclusion of her song being still gaping wide open. It would have been a much tamer and more decorous audience than we had in front that would not have tittered at this. I rushed for the scene shifter. He was not to be seen anywhere, nor did I see him again until the close of the performance, when I found him in one of the unused dressing rooms, in a sound sleep, with an empty flask in one hand and a half smoked clay pipe in the other. One of his assistants I found nervously reading the "plot" of the stage, and there the directions were plainly written to close in the angel with the flats in the first groove. He had conscientiously started to obey these instructions, and had run the flat square against the head of one of the captains of Cyrus's army, who lay sleeping there, ready to rise upon his cue and go on with the business that was set down for him. The assistant had therefore pulled back the flat, and more or less bewildered had gone for help to the "plot." I showed him what to do and where the mistake lay, and pushing one flat into its place, he ran around to the opposite side and pushed its mate in, finally closing in the angel, who came off from her pedestal and showed that she was of the earth, earthy, rather than of the heaven, heavenly, by falling into a hysterical fit of tears on account of the faux pas. I couldn't comfort her, for I sank into a chair almost overcome and rather wanting some comfort myself. Just then, however, the two wings of the army of Cyrus, in their brilliant uniforms, filed upon the stage from either side, and I felt that perhaps the stirring music and the intricate evolutions would atone for what had happened. From where I sat I could see most of the stage, and to my dismay I observed the big green bank upon which Cyrus had been reclining was still where it had been placed for him. The drill could not take place with that in the way, it would utterly impede the movements of the soldiers. I sprang from my chair and rushed upon the stage. Belshazzar's master of ceremonies daring the whole army of his bitterest enemy to drag out of its way a green bank! The inappropriateness of this movement on my part was only too apparent to the audience. They almost burst in the estacy of their merriment. I had forgotten, too, that the file leader of one detachment of these troops had been promoted, and was but little prepared for the complete failure of their drill. It became worse than a torchlight procession of firemen whose systems held as much benzine as their torches. The circle they were to have formed was turned into a very uncertain parabola, the cross became more like a pin-wheel in the midst of its most energetic revolutions; their "four fronts," "six fronts," "eight fronts," and "twelve fronts" were like tow strings thrown carelessly upon the ground, and their full front a good model for a bow. The captain in command, in his nervous excitement, knocked off his helmet with a movement of his sword, and before he could recover it it had rolled down to the footlights and over into the orchestra. And worse than all, right in the midst of the evolutions, the music stopped entirely. The female pianist had alone played the march, and understood that at a certain point the other instruments were to take up another one and relieve her. Coming to this place, she ceased playing, and nothing could be heard but the rather monotonous and regular tramp, tramp of the soldiers. The first violin, who led the other instruments, had not yet found his place, and when he did at length find it, what had been appointed to him to play was in another key from the preceding tune, and he struck in out of time with the soldiers. They were immediately thrown into confusion, and the effect was ludicrous in the extreme. Then, at the end of the march, when the soldiers were to have a grand chorus, there must, of course, be a return to the music of the Cantata. They marched down to the front in two lines and halted, ready to sing. The place in the score had to be found, amidst an oppressive silence, and the return to the key was as marked as had been the leaving of it. It was one of the happiest moments of my existence when the troops filed up the stage and the curtain came down.

For I was getting eager for the last act. First, because I wanted the whole thing to be concluded, and then I felt quite certain that nothing could happen that would be unpleasant or disagreeable. There was only one scene in it, and all of the people had been most thoroughly drilled in their several parts. I was nervously earnest in my desire that it should pass off well and leave such a pleasing impression that all which had gone beforehand might be forgotten. Alas! for my hopes.

The stage was full of people. Belshazzar was on his throne with his wife and mother. The Magi were in the background adoring their god Baal. All the lights were turned on full, and the scene was truly a striking and brilliant one. My spirits rose, and I was congratulating myself that after all this concluding part would crown the whole evening as a pronounced success. In the midst of the toast drinking to the King and Queen, with the flash of the golden goblets and the really inspiring music, I could again hear that titter in front. I ran to the prompt desk and looked over the whole stage. Everything seemed to be perfectly in order. I could see nothing, positively nothing at all out of the way, until happening, for it was merely by chance that I looked that way, happening to cast my eye up toward the back of stage to the place where the handwriting was to appear, just over the top of the flat I could see the bald head of the man who was stationed there to pull away the sheet that concealed the writing on the wall. I hardly thought it possible that he could be seen by the audience, for he seemed to be hidden by the flys, and for a moment I watched him. He moved along from side to side, making himself sure that his preparations were all complete, looking, for all the world, like a dark colored speckled egg floating on milk. He stopped in the center and slowly raised himself up to look over. His forehead, eyes, nose and mouth appeared in plain view, the border lights shining full upon him. His eyes were turned downward, as if he was looking upon the handwriting just below him. The audience caught him just at that moment, and he was received with a shout of laughter which was more vehement because, for a second or two, the feeling had been suppressed. The poor fellow looked in front and saw that, though before unconscious of it, he was in full view of the whole house. His exit was more impressive than had been his entrance, ducking himself down with a frightened expression on his face, and with just such a motion as that with which a jack-in-the-box disappears. It was some time before the business of the piece could go on.

Feeling as I did then, if I had had the power that Belshazzar is represented as being possessed of, I would have called rather for an executioner than an interpreter, and it was a peculiarly apt inquiry of a little girl, who hardly appreciated the situation, when she asked her mother, in quite an audible tone of voice:

"Mamma, what makes the people all laugh so when God is writing on the wall?"

The climax, in all ways, was reached at the conclusion of the piece. Cyrus rushes in with his troops and slays Belshazzar, who, surrounded by his people, falls dead upon the stage, amidst red fire and a great chorus outburst. That was what should have been. What took place was that Cyrus rushed in and ran his sword in the established way through Belshazzar. The poor king couldn't fall. If he stepped forward the curtain was in his way, and the people surrounding him so crowded upon him on every side that he couldn't go backward nor sideways. So the curtain came down when it should, with the Babylonish monarch standing upright, with the sword of his conqueror sticking through him. It was too much for the audience. Their laughter became shrieks, and I thought they would never cease and would never leave the hall.

The Erato Club never got over that Belshazzar. It was also their handwriting on the wall, the only comforting part about it being that it furnished them with sufficient funds to cancel all of their debts and honorably dissolve. As for me, my respect for any one who manages the stage for a play is almost unbounded.

